# The difference in pathogenic bacteria between chronic rhinosinusitis in patients with and without Sjogren’s syndrome: a retrospective case–control study

**DOI:** 10.1186/s12879-022-07652-4

**Published:** 2022-08-02

**Authors:** Pei-Rung Yang, Wei-Tai Shih, Yao-Hsu Yang, Chia-Yen Liu, Ming-Shao Tsai, Yao-Te Tsai, Cheng-Ming Hsu, Ching-Yuan Wu, Pey-Jium Chang, Geng-He Chang

**Affiliations:** 1 Department of Traditional Chinese Medicine, Chang Gung Memorial Hospital, Chiayi, Taiwan; 2 School of Traditional Chinese Medicine, Chang Gung University, College of Medicine, Taoyuan, Taiwan; 3 Health Information and Epidemiology, Laboratory of Chang Gung Memorial Hospital, Chiayi, Taiwan; 4 Department of Otolaryngology-Head and Neck Surgery, Chiayi Chang Gung Memorial Hospital, No. 6, W. Sec., Jiapu RD., Chia-Yi County, 61363 Puzi City, Taiwan; 5 Graduate Institute of Clinical Medical Sciences, College of Medicine, Chang Gung University, Taoyuan, Taiwan; 6 Faculty of Medicine, College of Medicine, Chang Gung University, Taoyuan, Taiwan; 7 Head and Neck Infection Treatment Center, Chang Gung Memorial Hospital, Chiayi, Taiwan

**Keywords:** Sinusitis, Bacteria, Pathogen, Sjogren’s syndrome, *Pseudomonas*, Chang Gung Research Database

## Abstract

**Background:**

Chronic rhinosinusitis (CRS) affects the quality of life of many people worldwide and can cause comorbidities. Our previous research proved that Sjogren’s syndrome (SS) is a predisposing factor for CRS, with a 2.5-fold associated risk. Antibiotics are important in CRS treatment; however, there is a paucity of research on the pathogenic bacteria of SS-CRS in the past. We conducted this study to investigate the pathogenic difference of SS-CRS and non-SS-CRS and aimed to give clinicians references when selecting antibiotics to treat SS-CRS.

**Materials and methods:**

A total of 14,678 patients hospitalized for CRS operation from 2004 to 2018 were identified from the Chang Gung Research Database. These CRS cases were classified as either SS-CRS or non-SS-CRS. We analyzed their bacterial distribution by studying the results of the pus cultures performed alongside surgery.

**Results:**

The top three facultative anaerobic or aerobic isolated bacteria in the SS-CRS group were coagulase-negative *Staphylococcus* (CoNS: 34.3%), *Pseudomonas aeruginosa* (28.6%), methicillin-sensitive *Staphylococcus aureus* (MSSA: 20%), and *Staphylococcus epidermidis* (20%). In the non-SS-CRS group, S. *epidermidis* (29.3%), CoNS (25.7%), and MSSA (14.2%) were identified. The top three anaerobic bacterial genera were *Cutibacterium* (54.3%), *Peptostreptococcus* (11.4%), and *Fusobacterium* (11.4%) in the SS-CRS group and *Cutibacterium* (53.8%), *Peptostreptococcus* (25%), and *Prevotella* (12.9%) in the non-SS-CRS group.

**Conclusions:**

*P.* *aeruginosa* is a major pathogen in SS-CRS patients. In addition, physicians should be aware of potential *Fusobacterium* and antibiotic-resistant bacterial infection in patients with SS-CRS.

**Supplementary Information:**

The online version contains supplementary material available at 10.1186/s12879-022-07652-4.

## Background

Chronic rhinosinusitis (CRS), which affects 5–12% of the general population worldwide [1–5], is a common chronic inflammatory condition with a high incidence and refractory symptoms such as rhinorrhea, nasal congestion, post-nasal discharge, and cough [6]. In addition to focal sinonasal symptoms such as facial discomfort and anosmia, CRS causes significant adverse effects on the quality of life, particularly in physical and social functioning [7].

Standard treatments for CRS include medications, such as oral antibiotics and steroids and endoscopic sinus surgery [1]. Moreover, various adjuvant therapies are used to control chronic sinus symptoms, including topical steroids, nasal irrigation with or without drugs, steam inhalation, and leukotriene antagonists [8]. It is estimated that patients with CRS worldwide will lose approximately $13–60 billion annually in direct and indirect associated costs [9] and will miss an average of 18.7 days of work per patient per year [10].

Previous studies have found that patients with autoimmune diseases such as rheumatoid arthritis (RA) and systemic lupus erythematosus are predisposed to CRS [11]. Some reports indicated that Sjogren’s syndrome (SS), a chronic autoimmune disease characterized by lymphocyte infiltration of exocrine glands, particularly the salivary and lacrimal glands, which could cause xerostomia and keratoconjunctivitis sicca [12], involves inflammation and infection of the respiratory tract [13]. Approximately 10–20% of patients with SS suffer from interstitial lung disease [14], and there is a potential risk of community-acquired pneumonia and interstitial pneumonia [15]. Moreover, SS may affect both the lower and upper respiratory tracts. Without natural tears from the nasolacrimal duct, sinus duct secretion decreases, the sinus cavity becomes drier, and an increase in mucus viscosity may result in impaired mucociliary function [16]. Therefore, SS can cause upper respiratory tract infection and local sinus inflammation more frequently, and predisposes patients to CRS. A relationship between sicca symptoms and CRS was reported [13], and we previously demonstrated using a national database that patients with SS are 2.5 times more susceptible to CRS than those without SS [17].

In general, CRS is classified into CRS with nasal polyps (CRSwNP) or CRS without nasal polyps (CRSsNP) phenotypically [1]. CRSwNP is often characterized by T helper type 2 (Th2) cytokine expression, which is thought to be an inflammatory etiology, whereas infection is often responsive for the main etiology of CRSsNP [18]. Our previous study demonstrated that SS patients’ CRS tend to be CRSsNP and the recurrent infections of SS-CRS might be related to the poor mucociliary function and the impaired local immunity of the sinonasal cavity [17]. The dysbiosis of sinonasal microbiota might be fostered by exaggerated host immunity and the over-growth of disease-associated bacteria could play an important role in the pathogenesis of SS-CRS [19, 20]. Because CRS accounts for approximately 1–2% of total physician visits and is associated with significant comorbidities, and dangerous fetal complications such as orbital cellulitis, intracranial abscesses, and subperiosteal abscesses that increase the mortality rate [21, 22], appropriate drug treatments for chronic sinusitis must be administered to reduce the risk of acute exacerbation of inflammation [9]. Therefore, comprehensively identifying the bacterial distribution of CRS may aid in the selection of appropriate empirical antibiotics for CRS treatment, which could be helpful for clinical therapeutic efficiency. Previous studies have found that *Staphylococcus aureus* and anaerobes such as *Prevotella*, *Bacteroides* species (sp.), and *Peptostreptococcus* sp. are the most common CRS pathogens in the general population [23–26]. Amoxicillin–clavulanate is typically recommended as the first-line regimen for most patients with CRS, with alternatives including clindamycin, metronidazole combinations with a second- or third-generation cephalosporin, macrolides, or trimethoprim–sulfamethoxazole [27–29]. Those findings from the previous research investigating the bacteria strains of CRS provided an important reference for clinicians to select empiric antibiotics for CRS treatment. However, to date, there has been a paucity of studies that explore the bacterial strains in patients with CRS who also suffer from SS. The null-hypothesis tested was that CRS patients with SS do not have different bacterial compositions as compared to non-SS-CRS patients. Therefore, we conducted this study by using the Chang Gung Multi-institutional Database to explore the bacterial difference of CRS in patients with and without SS and investigated important and valuable information about the clinical management of SS-CRS.

## Materials and methods

### Data source: the Chang Gung Research database (CGRD)

This study used CGRD, which contains identified data collected from the patients’ medical records at Chang Gung Memorial Hospital (CGMH), and these data are systematically updated each year to generate new data at the CGMH. The CGMH was established in 1976 and is currently the largest healthcare system in Taiwan, comprising seven medical institutions located in the northeast to the south of Taiwan, with 10,070 beds, and accommodating over 280,000 patients annually [30]. Certain studies, including a multicenter study with a relatively large sample size, were based on CGRD.

We conducted research according to the guiding principles of the Declaration of Helsinki. The requirement for participants’ informed consent was waived because all the data collected were de-identified and the study did not infringe on their rights or negatively affect their welfare. The waived of informed consent was provided by the committee of Chang Gung Medical Foundation Institutional Review Board. The Ethics Review Board of Chang Gung Memorial Hospital, Chia-Yi Branch, Taiwan, approved this study with serial number 201900347B0.

### Study population

**Fig. 1:**
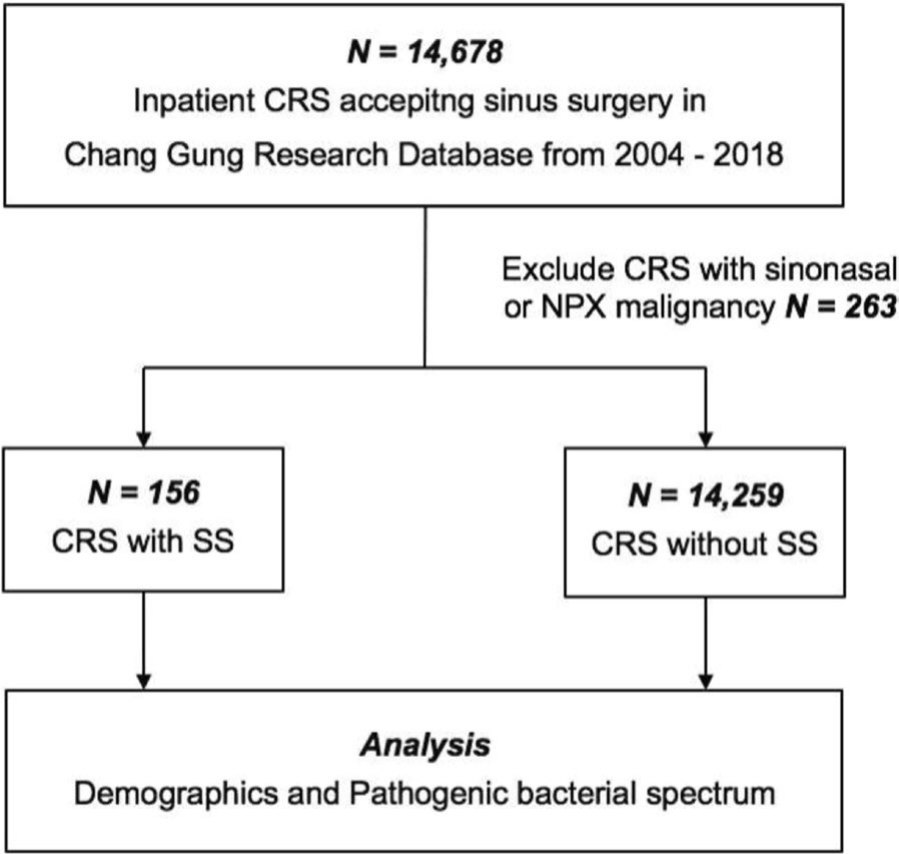
Enrollment of inpatient with CRS-operation. *CRS* chronic rhinosinusitis, *NPX* nasopharynx, *SS* Sjogren’s syndrome

Hospitalized patients who were diagnosed with CRS related International Classification of Diseases, Ninth or Tenth Revision, Clinical Modification (ICD-9-CM code: 473 and ICD**-**10-CM code: J32) and accepted to undergo sinus surgery were identified from the CGRD (01.01.2004–12.31.2018), which comprised 14,678 patients. We excluded patients with malignant tumors, including sinonasal or nasopharyngeal cancer according to ICD-9-CM codes. Further, we used ICD codes of SS (ICD-9: 710.2 and ICD-10: M35.00, M35.01, and M35.09) to separate the CRS cases into two groups: CRS in patients with SS (study group) and CRS in patients without SS (control group). We analyzed the results of bacterial culture performed during sinusitis surgery in those patients with CRS from CGRD and investigated the difference in bacterial compositions between SS-CRS and non-SS-CRS (Fig. 1).

### Comorbidities

Comorbidities of the CRS cases included RA (ICD-9-CM code: 714), diabetes mellitus (DM) (ICD-9-CM code: 250), hypertension (HTN) (ICD-9-CM codes: 401–405), chronic kidney disease (CKD) (ICD-9-CM codes: 585, 586, 403, and 404), cerebrovascular accident (CVA) (ICD-9-CM codes: 430–438), coronary artery disease (CAD) (ICD-9-CM codes: 410–414), chronic obstructive pulmonary disease (COPD) (ICD-9-CM codes: 491, 492, and 496), and asthma (ICD-9-CM code: 493). Medical comorbidities were included if they appeared one or more times in the diagnoses of inpatients and three or more times in the diagnoses of outpatients within 12 months before the index date of sinus surgery for CRS.

### Bacterial distribution

Facultative anaerobes such as *Staphylococcus* sp., *Klebsiella* sp., *Streptococcus* sp. and aerobes such as *Pseudomonas* sp. are considered as major pathogenic bacteria for CRS, and empirical antibiotics typically target these pathogens. Therefore, we classified the bacteria identified in patients with CRS during surgery into three groups: facultative anaerobes or aerobes, anaerobes, and fungi, and calculated the proportions of these bacteria in the SS-CRS and non-SS-CRS groups.

### Statistical analysis

The sociodemographic profiles of patients with CRS with and without SS were analyzed using Pearson’s chi-squared test. All data were processed and analyzed using SAS version 9.4 (SAS Institute Inc., Cary, NC, USA), and the threshold for statistical significance was set at *p* < 0.05.

## Results

### Characteristics of patients in the SS-CRS and non-SS-CRS groups

From January 1, 2004 to December 31, 2018, a total of 14,678 patients hospitalized for CRS surgery were identified from the CGRD. Two hundred sixty-three patients with sinusitis were excluded because of comorbid sinonasal or nasopharyngeal cancer. A total of 156 CRS patients with SS (SS-CRS) and 14,259 CRS patients without SS (non-SS-CRS) were enrolled in the study (Fig. 1). The characteristics of the study and control groups are summarized in Table 1. There were significantly higher proportions of female patients (72.4% vs. 39.3%, *p* < 0.001) and medical comorbidities such as RA (12.2% vs. 0.5%, *p* < 0.001), DM (15.4% vs. 9.3%, *p* = 0.009), HTN (40.4% vs. 18.0%, *p* < 0.001), CKD (9.6% vs. 1.8%, *p* < 0.001), CVA (10.3% vs. 3.1%, *p* < 0.001), CAD (10.3% vs. 3.8%, *p* < 0.001), COPD (16.7% vs. 5.7%, *p* < 0.001), and asthma (11.5% vs. 6.8%, *p* = 0.021) in the SS-CRS group than in the non-SS-CRS group.

**Table 1:** Demographic characteristics of SS-CRS and non-SS-CRS

Variables	CRS-SS		CRS-Non-SS		
	N = 156		N = 14,259		*p*-value^*^
	n	%	n	%	
Gender					< 0.001
Male	43	27.6	8653	60.7	
Female	113	72.4	5606	39.3	
Age (years)					0.001
< 65	120	76.9	12,348	86.6	
≥ 65	36	23.1	1911	13.4	
Covariates					
RA	19	12.2	68	0.5	< 0.001
DM	24	15.4	1325	9.3	0.009
HTN	63	40.4	2562	18.0	< 0.001
CKD	15	9.6	261	1.8	< 0.001
CVA	16	10.3	441	3.1	< 0.001
CAD	16	10.3	545	3.8	< 0.001
COPD	26	16.7	807	5.7	< 0.001
Asthma	18	11.5	973	6.8	0.021

*CRS* chronic rhinosinusitis, *SS* Sjogren’s syndrome, *RA* rheumatoid arthritis, *DM* diabetes mellitus, *HTN* hypertension, *CKD* chronic kidney disease, *CVA* cerebral vascular accident, *CAD* coronary artery disease, *COPD* chronic obstructive pulmonary disease

^*^Pearson’s Chi-square tests

### Bacterial distribution

Bacterial culture data were obtained when the patients underwent sinus surgery. The bacterial cultures were performed in 25.6% of patients with SS-CRS and 27.5% of patients with non-SS-CRS, and the positive culture rates were 87.5% and 91.2%, respectively. Based on the culture results, the bacteria were classified into (1) facultative anaerobes or aerobes, (2) anaerobes, and (3) fungi. The details of the culture results are provided in the supplementary data (Additional file 1: Table S1).

### The compositions of isolated bacterial infections

**Fig. 2:**
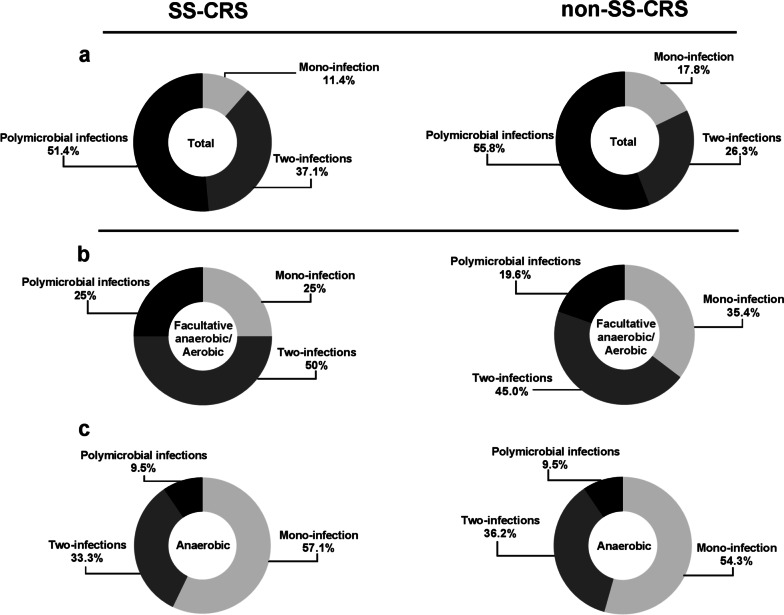
The compositions of isolated bacterial species in SS-CRS and non-SS-CRS. **a** Total species. **b** Facultative anaerobic or aerobic species. **c** Anaerobic species

We analyzed the compositions of bacterial infections of each patient with SS-CRS and non-SS-CRS, and classified the results into three groups: mono-microbial, two-microbial and poly-microbial infections. There was an average of 2.4 ± 0.7 of isolated bacteria grown from one individual patient in SS-CRS and 2.4 ± 0.8 in non-SS-CRS. Poly-microbial infections (51.4%) were the majority followed by two-microbial infections (37.1%) and mono-microbial infections (11.4%) in whole isolated bacteria in SS-CRS and poly-microbial infections (55.8%), two-microbial infections (26.3%) and mono-microbial infections (17.8%) in non-SS-CRS, respectively (Fig. 2a). Furthermore, the compositions of isolated bacterial infections of each patient with SS-CRS and non-SS-CRS of facultative anaerobes or aerobes and anaerobes separately were showed in Fig. 2b, c.

### Most frequently isolated taxa: genus level

**Fig. 3 A:**
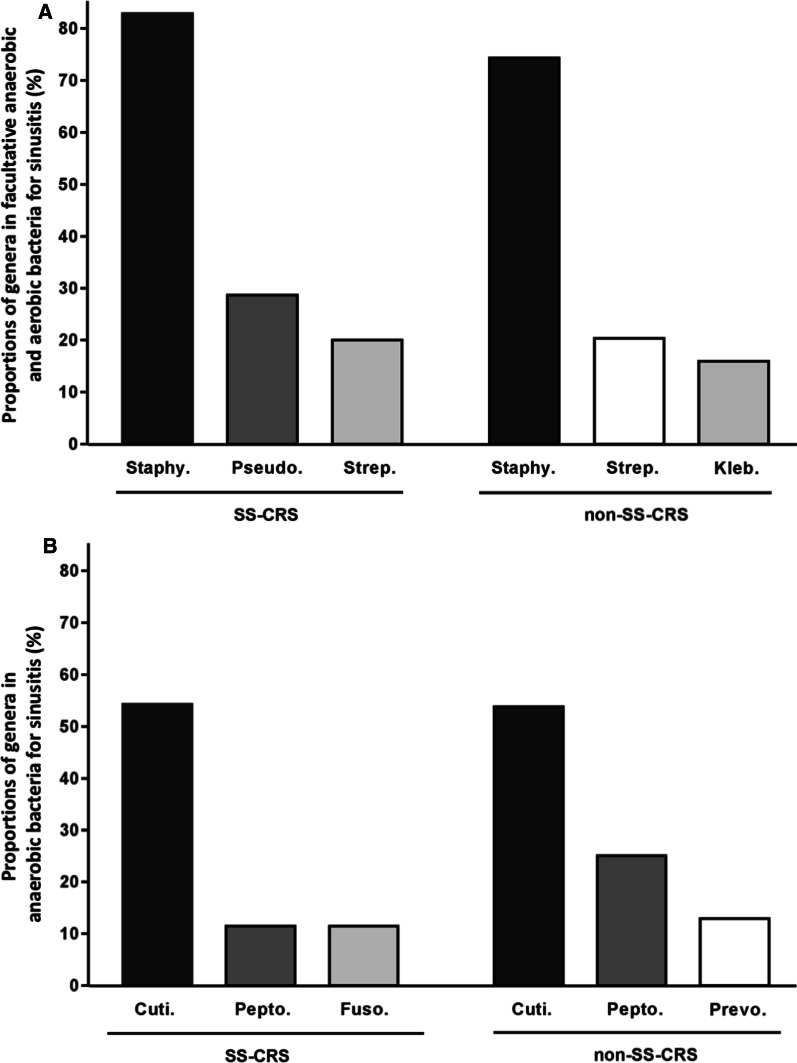
Top three bacterial genera of facultative anaerobes or aerobes in SS-CRS and non-SS-CRS. Staphy., *Staphylococcus*; Pseudo., *Pseudomonas*; Strep., *Streptococcus*; Kleb., *Klebsiella*; SS, Sjogren’s Syndrome; CRS, chronicrhinosinusitis. **B** Top three bacterial genera of anaerobes in SS-CRS and non-SS-CRS. Cuti., *Cutibacterium*; Pepto., *Peptostreptococcus*; Fuso., *Fusobacter*; Prevo., *Prevotella*

The three major bacterial genera responsible for SS-CRS of facultative anaerobes or aerobes were *Staphylococcus* (82.9%), *Pseudomonas* (28.6%), and *Streptococcus* (20.0%); the three for non-SS-CRS were *Staphylococcus* (74.3%), *Streptococcus* (20.3%), and *Klebsiella* (15.9%) (Fig. 3A). The three most frequently encountered anaerobic genera in SS-CRS were *Cutibacterium* (54.3%), *Peptostreptococcus* (11.4%), and *Fusobacterium* (11.4%); the three in non-SS-CRS were *Cutibacterium* (53.8%), *Peptostreptococcus* (25%), and *Prevotella* (12.9%) (Fig. 3B).

Yeast infection was found in 2.9% of SS-CRS, whereas for non-SS-CRS, it was 0.9% mold, 0.8% *Aspergillus*, and 0.5% *Candida*.

### Most frequently isolated taxa: species level

**Fig. 4 A:**
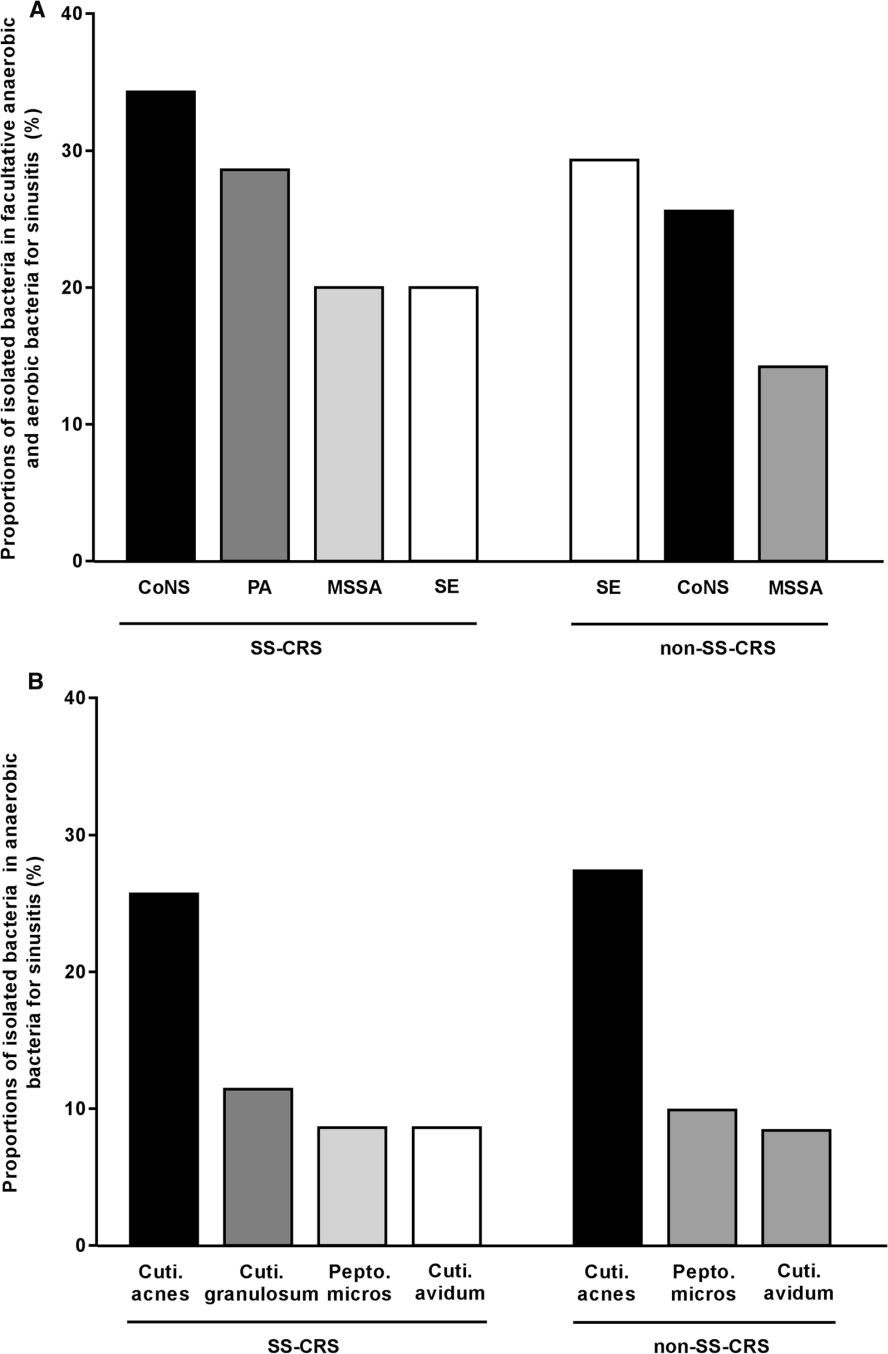
Top three bacterial species of facultative anaerobes or aerobes in SS-CRS and non-SS-CRS. CoNS, Coagulase-negative *Staphylococcus*; PA, *Pseudomonas aeruginosa*; MSSA, Methicillin-sensitive *Staphylococcus aureus*; SE, *Staphylococcus epidermidis.* **B** Top three bacterial species of anaerobes in SS-CRS and non-SS-CRS. Cuti., *Cutibacterium*; Pepto., *Peptostreptococcus*

In the speculum of facultative anaerobes or aerobes, the three major isolated bacteria in SS-CRS were coagulase-negative *Staphylococcus* (CoNS) (34.3%), *Pseudomonas aeruginosa* (28.6%), and methicillin-sensitive S. *aureus* (MSSA) (20%), and *Staphylococcus epidermidis* (20%); the three for non-SS-CRS were S. *epidermidis* (29.3%), CoNS (25.6%), and MSSA (14.2%) (Fig. 4A). The three most frequently encountered anaerobes in SS-CRS were *Cutibacterium acnes* (25.7%), *Cutibacterium granulosum* (11.4%), *Peptostreptococcus micros* (8.6%), and *Cutibacterium avidum* (8.6%); the three in non-SS-CRS were C. *acnes* (27.4%), C. *avidum* (9.9%), and P. *micros* (8.4%) (Fig. 4B).

### Antibiotic-resistant bacteria

**Fig. 5:**
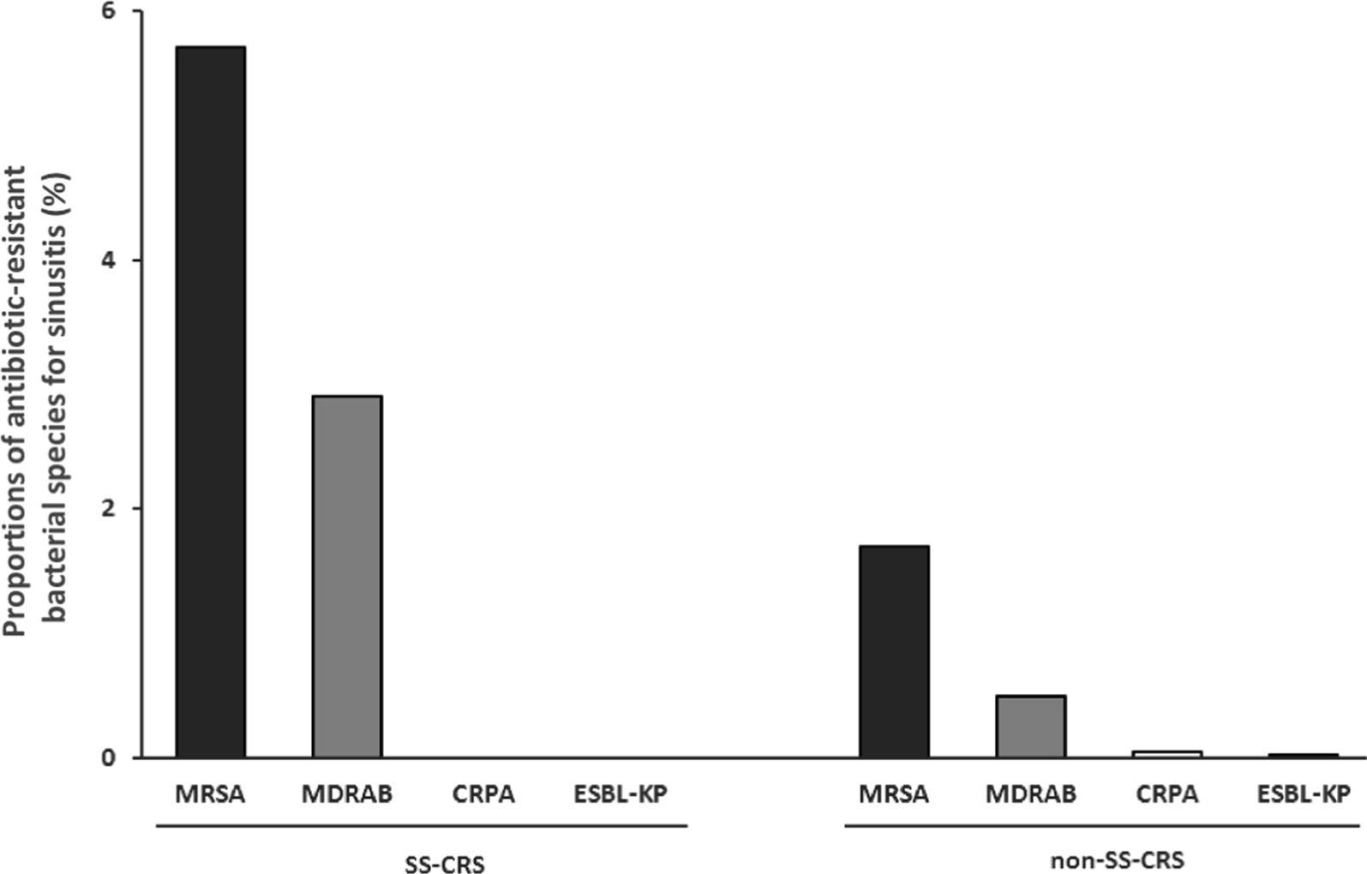
The major antibiotic-resistant bacterial species in SS-CRS and non-SS-CRS. MRSA, Methicillin-resistant *Staphlococcus aureus*; extended-spectrum β-lactamases producing *Klebsiella pneumoniae* (ESBL-KP); carbapenem-resistant *Pseudomonas aeruginosa* (CRPA); multidrug-resistant *Acinetobacter baumannii* (MDRAB)

Based on bacterial culture results, the major antibiotic-resistant bacteria in clinical practice were further analyzed. These included Methicillin-resistant S. *aureus* (MRSA), extended-spectrum β-lactamases producing *Klebsiella pneumoniae* (ESBL-KP), carbapenem-resistant P. *aeruginosa* (CRPA), and multidrug-resistant *Acinetobacter baumannii* (MDRAB). The percentages of these drug-resistant pathogens in the SS-CRS group were MRSA (5.7%), MDRAB (2.9%), CRPA (0%) and ESBL-KP (0%) and those in the non-SS-CRS group were MRSA (1.7%), MDRAB (0.5%), CRPA (0.06%) and ESBL-KP (0.03%) (Fig. 5).

## Discussion

In our previous national study, SS was proven to be a risk factor for CRS [17]. According to our findings, SS-CRS is predominantly sinusitis without polyposis, and the secretions of patients with SS-CRS have been clinically observed to be yellow and sticky. Precise oral antibiotic administration or nasal irrigation combined with antibiotic treatment could help improve therapeutic efficiency [31–33].

Poly-microbial infections were the majority in either SS-CRS or non-SS-CRS in our study. This results were consistent with the findings of previous studies analyzing the bacterial compositions of CRS [34, 35]. A previous study reported the number of bacteria identified from pus cultures in patients with CRS ranged from two to five [35] and another study discovered an average of 3.4 kinds of bacteria isolated from each CRS patients [34]. Our study demonstrated that the average number of isolated bacteria grown from one individual patient was 2.4 in SS-CRS and non-SS-CRS. Besides, the compositions of bacterial infections of each patient in the speculum of facultative anaerobes or aerobes and anaerobes with SS-CRS and non-SS-CRS were not significantly different.

Comparing the results of previous studies with our findings, our study analyzed the bacterial species of a large number of patients with sinusitis, and the results were consistent with the previous studies, all of which were dominated by poly-microbial infections, and the data was similar. It means that the results of our database-research are reliable, and the results of bacterial cultures in our study were obtained from specimens during sinusitis surgery, which can improve the accuracy of studying the bacterial species of CRS. In addition, the bacterial compositions of SS-CRS and non-SS-CRS, whether compared with all bacteria, or when facultative anaerobes or aerobic bacteria and anaerobes were compared separately, the results were similar between the two groups (Fig. 2), indicating that the SS patients’ sinusitis is not different from the non-SS patients’ sinusitis in terms of bacterial composition. Therefore, on this basis, our study analyzing the differences in bacterial species between the two groups would not be confounded by a different bacterial composition.

Observing the compositions of facultative anaerobic or aerobic bacteria in SS-CRS and non-SS-CRS, it can be found that two-infections accounted for the highest proportion (50% for SS-CRS; 45% for non-SS-CRS). Because facultative anaerobic or aerobic bacteria is generally the main target for our clinicians to think about the use of antibiotics. According to the findings of the bacterial spectrum of CRS in our study, when the therapeutic effect on CRS is not optimal and no clear information of bacterial infection can be obtained, it is necessary for clinicians to know which possible bacteria should be considered as an alternative target for treatment of CRS. In addition, further observation of the composition of anaerobic bacteria in SS-CRS and non-SS-CRS can find the mono-infection was the main type of anaerobic bacterial infection in patients with CRS (57.1% for SS-CRS; 54.3% for non-SS-CRS). This finding highlights the importance of our study, because anaerobic bacteria are generally not the main target of empiric antibiotics, but without effective treatment, sinusitis may persist or recur. Therefore, the findings of this study can provide clinicians an important information on thinking about an adequate combination of antibiotics for treating CRS.

In this study, the most common bacterial genus responsible for CRS in patients without SS was *Staphylococcus* followed by *Streptococcus*. Our study’s control group results were consistent with those of several previous studies [20, 23, 36, 37], and this indirectly reflected that the bacterial spectra identified in our study to be responsible for SS-CRS were highly reliable.

The primary finding of our study was that the causative bacteria in SS-CRS differed from those in non-SS-CRS. Based on the results of this study, the null-hypothesis of the present study that CRS patients with SS do not have different bacterial compositions as compared to non-SS-CRS patients was declined. With respect to the main identified bacteria, non-SS-CRS was *S*. *epidermidis* (29.3%) and then CoNS (25.6%), and SS-CRS was CoNS (34.3%) and then *P*. *aeruginosa* (28.6%). CoNS is generally considered to be the bacteria that constitutes the commonly encountered flora in the nasal cavity, and the choice of empirical antibiotics is generally similar to that used in the treatment of *S*. *epidermidis*. However, in patients with SS-CRS, *P.* *aeruginosa* plays a prominent role. Therefore, for patients with SS-CRS, the choice of antibiotics is important in the treatment of *P.* *aeruginosa* infection. *P*. *aeruginosa* was uncommonly found in the nasal culture of normal individuals, and, in general, the isolation of *P*. *aeruginosa* from symptomatic patients indicates that it is pathogenic and a potential cause of CRS [38]. *P*. *aeruginosa* has been shown to be a common pathogen in patients with CRS among special populations, including patients in intensive care units and those with immunodeficiency, DM, and mucociliary dysfunction such as cystic fibrosis [39]. *P*. *aeruginosa* was also found to be more common in patients receiving systemic steroids [40]. Thus, it may explain this study’s finding that *P*. *aeruginosa* was the commonly isolated bacterial species in SS-CRS. However, *P*. *aeruginosa*-related CRS is considered difficult to treat, as it can secrete various virulence factors and organize a tough biofilm to escape the immune system and destroy host cells, developing antibiotic resistance [41]. Therefore, *P*. *aeruginosa*-CRS must be treated with appropriate antibiotics.

*S*. *aureus* colonizes 20–30% of the nasal mucosa of normal individuals and is more common in patients with CRS [42]. It is known that S. *aureus* toxin-producing strains are effective disease modifiers that can destroy barrier function, invade epithelial cells, regulate immune cells, and promote polyp formation [43]. Although S. *aureus* is the main pathogen in CRS, most species are still MSSA [44], as our study showed. However, the proportion of MRSA identified from bacterial cultures was higher in the SS-CRS group than in the non-SS-CRS group (MRSA: SS-CRS vs. non-SS-CRS: 5.7% vs. 1.7%) (Additional file 1: Table S1). Research on MRSA nasal colonization has recently increased [45], and MRSA is now known to cause slow mucosal healing and infection at the surgical site and biofilm formation [46]. Biofilm formation, associated with increased disease severity, is a response to selective pressure in the mucosal niche, including the use of antibiotics and loss of integrity of the host epithelial immune barrier, which may lead to a greater overall morbidity [47].

A. *baumannii*, an aerobic Gram-negative coccobacilli, is a significant bacteria that can cause severe and recurrent infections with consequent morbidities [48]. This pathogen is usually identified in immunocompromised individuals or patients with comorbidities or chronic illnesses such as prolonged hospital stays, poorly controlled DM, or malignancies, et al. [49]. *A*. *baumannii*, which can effectively evade the effects of antibacterial drugs, rapidly develops resistance to antimicrobials, and a high incidence of multidrug-resistant strains (MDRAB) has been identified [48]. In this study, the SS-CRS group had a higher proportion of patients with MDRAB-induced sinonasal infection than the non-SS-CRS group (Fig. 5). This finding could provide clinicians with important information, and when encountering sinusitis that is resistant to medical treatment, the possibility of infection caused by drug-resistant bacteria should be considered.

The common genera of anaerobes in CRS were *Cutibacterium* and *Peptostreptococcus* [36] as shown in this study. *Fusobacterium* was another major pathogen in SS-CRS, which differed from those identified in non-SS-CRS. Anaerobic bacteria typically influence antibiotic selection to include anaerobic bacterial coverage; therefore, this finding is essential for promoting effective SS-CRS management. *Fusobacterium* are known to have mobile genetic components that increase their antibiotic resistance and virulence with other opportunistic bacteria in the community, creating a pathogenic niche [50]. Moreover, *Fusobacterium* may cause serious CRS complications, such as intracranial abscesses [21]. Therefore, in patients with SS-CRS, *Fusobacterium* infection may be latent, necessitating prompt and appropriate antibiotic treatment.

Our study has several strengths. We identified up to 14,678 CRS cases from the CGRD database, which comprised more than 14% of inpatient coverage of Taiwan [30], and the characteristics of the study population were similar to those of the population in our previous population-based nationwide study in Taiwan [17]; therefore, the results of this study could present real-world evidence. In addition, the Registry of Catastrophic Illness Patients Database affiliated with the National Health Insurance Research Database was used to confirm the SS diagnoses of the patients and to confirm their CRS diagnoses depending not only on CRS-related ICD-9 codes but also on surgery codes specifically by otolaryngologists. It provided accurate and reliable patient data.

In addition, the strong point of this research was that the bacterial cultures of the sinusitis cases were based on the pus obtained during surgical operations. They differed from the bacterial cultures of the nasal secretions collected in the outpatient clinic. These bacterial culture results could be more accurate and better represent the actual pathogenic bacteria of sinusitis. Although we only collected data on the results of sinusitis strains of patients who underwent surgery, it could not represent the status of all sinusitis cases; however, patients who need surgery are generally considered to have more serious or refractory disease manifestation and are clinically difficult to treat. In our study, the analysis of the bacterial species responsible for sinusitis in patients who agreed to undergo surgery was beneficial to clinicians for subsequent reference for the selection of antibiotics.

Our study also has some limitations. For patients with sinusitis, most of the bacterial culture results varied. Nevertheless, we added up the proportion of each bacterial species and compared the difference between SS-CRS and non-SS-CRS, but in fact, the bacterial compositions in patients with CRS were complicated. In addition, age and other comorbidities may be a source of potential bias that could affect the results. Although, the demographic characteristics and comparison of bacterial growth of SS-CRS and non-SS-CRS (Additional file 2: Table S2 and Additional file 3: Table S3) as well as the percentages of top three bacterial genera and species of facultative anaerobes or aerobes and anaerobe in the age of SS-CRS and non-SS-CRS (Additional files 4 and 5: Figs. S1 and S2) were investigated, but the numbers of SS-CRS used for bacterial composition analysis were insufficient to provide matching data on these confounding factors. In the follow-up study, more data for predictive analysis of age and other system diseases and the relationship between the disease severity and pathogenic bacteria as well as the linkage between the bacteria and SS disease activity, and the changes in trends of bacteria, and their antibiotic resistance over time are all areas worthy of research and discussion.

## Conclusions

P. *aeruginosa* is a major aerobic pathogen in SS-CRS that is different from patients with non-SS-CRS. As for anaerobic bacteria, *Fusobacterium* infection should be paid attention to in patients with SS-CRS. In addition, drug-resistant pathogens containing MRSA and MDRAB, accounted for a higher proportion of SS-CRS than of non-SS-CRS. The above findings on the characteristics of pathogenic bacteria for SS-CRS could provide clinicians with important references when selecting antibiotics to treat CRS.

## Supplementary Information

**Additional file 1: Table S1. **Bacterial difference of SS-CRS versus non-SS-CRS.

**Additional file 2: Table S2. **Demographic characteristics in bacterial growth of SS-CRS and non-SS-CRS.

**Additional file 3: Table S3. **The comparison of bacterial genera between SS-CRS and non-SS-CRS.

**Additional file 4.** **Figure S1.** Top three bacterial genera of facultative anaerobes or aerobes and anaerobe in the age of SS-CRS and non-SS-CRS.

**Additional file 5.** **Figure S2.** Top three isolated bacteria of facultative anaerobes or aerobes and anaerobe in the age of SS-CRS and non-SS-CRS.

## Data Availability

If detailed data are required, they can contact the Correspondence of the study, Geng-He Chang (Email address: genghechang@gmail.com).

## Abbreviations

CRS: Chronic rhinosinusitis
SS: Sjogren’s syndrome
CoNS: Coagulase-negative *staphylococcus*
MSSA: *Methicillin-sensitive Staphylococcus aureus*
MRSA: *M* *ethicillin-resistant Staphylococcus aureus*
S. *epidermidis*: *Staphylococcus epidermidis*
*sp*.: Species
S. *aureus*: *Staphylococcus aureus*
CGRD: Chang Gung Research Database
CGMH: Chang Gung Memorial Hospital
ICD-9-CM: International Classification of Diseases, Ninth Revision Clinical Modification
RA: Rheumatoid arthritis
DM: Diabetes mellitus
HTN: Hypertension
CKD: Chronic kidney disease
CVA: Cerebrovascular accident
CAD: Coronary artery disease
COPD: Chronic obstructive pulmonary disease
ESBL-KP: Extended-spectrum β-lactamases producing *Klebsiella **pneumoniae*
CRPA: Carbapenem-resistant *Pseudomonas aeruginosa*
MDRAB: Multidrug-resistant *Acinetobacter baumannii*
*A.**baumannii*: *Acinetobacter* *baumannii*
