# Supplementary material for: The difference in pathogenic bacteria between chronic rhinosinusitis in patients with and without Sjogren’s syndrome: a retrospective case–control study
Source: BMC Infect Dis. 2022 Aug 2;22:666. doi: 10.1186/s12879-022-07652-4 (PMC9344658; doi:10.1186/s12879-022-07652-4)
Supplement: Supplementary file 1 — Additional file 1: Table S1. Bacterial difference of SS-CRS versus non-SS-CRS. [file 12879_2022_7652_MOESM1_ESM.docx]

| **Additional Table S1. Bacterial difference of SS-CRS versus non-SS-CRS** | | | | | | |
| --- | --- | --- | --- | --- | --- | --- |
|  |  |  | | |  | |
|  |  | **SS-CRS** | | | **non-SS-CRS** | |
|  |  | **N** | **%** | **N** | | **%** |
| **Cases** |  | 156 |  | 14259 | |  |
| Bacterial cultures |  | 40 | 25.6 | 3920 | | 27.5 |
| Bacterial growth |  | 35 | 87.5 | 3575 | | 91.2 |
| **Classification** |  | n | % | n | | % |
| **F. anaerobic^*^ / GPC** | ***Staphylococcus*** | **29** | **82.9** | **2657** | | **74.3** |
|  | *Staphylococcus aureus* | 9 | 25.7 | 566 | | 15.8 |
|  | Methicillin-resistant (MRSA) | 2 | 5.7 | 59 | | 1.7 |
|  | Methicillin-sensitive (MSSA) | 7 | 20.0 | 507 | | 14.2 |
|  | *Staphylococcus epidermidis* | 7 | 20.0 | 1046 | | 29.3 |
|  | Coagulase (-) *staphylococcus* | 12 | 34.3 | 915 | | 25.6 |
|  | *Staphylococcus saccharolyticus* |  |  | 15 | | 0.4 |
|  | *Staphylococcus haemolyticus* |  |  | 37 | | 1.0 |
|  | *Staphylococcus capitis* |  |  | 10 | | 0.3 |
|  | *Staphylococcus pseudintermedius* |  |  | 1 | | 0.0 |
|  | *Staphylococcus hominis* |  |  | 5 | | 0.1 |
|  | *Staphylococcus lugdunensis* | 1 | 2.9 | 58 | | 1.6 |
|  | *Staphylococcus saprophyticus* |  |  | 4 | | 0.1 |
| **F. anaerobic / GNB** | ***Klebsiella*** | **5** | **14.3** | **568** | | **15.9** |
|  | *Klebsiella pneumoniae* | 3 | 8.6 | 489 | | 13.7 |
|  | *Klebsiella pneumoniae*-ESBL^#^ |  |  | 1 | | 0.0 |
|  | *Klebsiella oxytoca* | 1 | 2.9 | 75 | | 2.1 |
|  | *Klebsiella* sp. | 1 | 2.9 | 3 | | 0.1 |
| **F. anaerobic / GPC** | ***Streptococcus*** | **7** | **20.0** | **725** | | **20.3** |
|  | *Viridans streptococcus* | 3 | 8.6 | 307 | | 8.6 |
|  | *Streptococcus agalactiae* |  |  | 14 | | 0.4 |
|  | *β-streptococcus* non-ABD |  |  | 74 | | 2.1 |
|  | *β-streptococcus* group D |  |  | 3 | | 0.1 |
|  | *β-streptococcus* group F |  |  | 1 | | 0.0 |
|  | *Streptococcus pneumoniae* | 1 | 2.9 | 101 | | 2.8 |
|  | *Streptococcus anginosus* |  |  | 19 | | 0.5 |
|  | *Streptococcus constellatus* | 1 | 2.9 | 90 | | 2.5 |
|  | *Streptococcus oralis* | 1 | 2.9 | 18 | | 0.5 |
|  | *Streptococcus gordonii* |  |  | 2 | | 0.1 |
|  | *Streptococcus mitis* |  |  | 35 | | 1.0 |
|  | *Streptococcus intermedius* | 1 | 2.9 | 11 | | 0.3 |
|  | *Streptococcus dysgalactiae* |  |  | 1 | | 0.0 |
|  | *Streptococcus parasanguinis* |  |  | 4 | | 0.1 |
|  | *Streptococcus salivarius* |  |  | 44 | | 1.2 |
|  | *Streptococcus* sp. |  |  | 1 | | 0.0 |
| **F. anaerobic / GPC** | ***Enterococcus*** | **1** | **2.9** | **29** | | **0.8** |
|  | *Enterococcus faecalis* | 1 | 2.9 | 22 | | 0.6 |
|  | *Enterococcus faecium* |  |  | 1 | | 0.0 |
|  | *Enterococcus avium* |  |  | 1 | | 0.0 |
|  | *Enterococcus raffinosus* |  |  | 2 | | 0.1 |
|  | Enterococcus sp. |  |  | 3 | | 0.1 |
| **F. anaerobic / GNB** | ***Enterobacter*** | **2** | **5.7** | **352** | | **9.8** |
|  | *Enterobacter aerogenes* | 1 | 2.9 | 269 | | 7.5 |
|  | *Enterobacter cloacae* | 1 | 2.9 | 68 | | 1.9 |
|  | *Enterobacter gergoviae* |  |  | 7 | | 0.2 |
|  | *Enterobacter* sp. |  |  | 8 | | 0.2 |
| **F. anaerobic / GNB** | **E*.coli*** | **1** | **2.9** | **104** | | **2.9** |
|  | E*.coli* | 1 | 2.9 | 102 | | 2.9 |
|  | E*.coli*-ESBL |  |  | 2 | | 0.1 |
| **F. anaerobic / GNB** | ***Proteus*** | **1** | **2.9** | **76** | | **2.1** |
|  | *Proteus mirabilis* | 1 | 2.9 | 68 | | 1.9 |
|  | *Proteus vulgaris* |  |  | 8 | | 0.2 |
| **F. anaerobic / GNB** | ***Haemophilus*** |  |  | **149** | | **4.2** |
|  | *Haemophilus parainfluenzae* |  |  | 19 | | 0.5 |
|  | *Haemophilus influenzae* |  |  | 126 | | 3.5 |
|  | *Haemophilus haemolyticus* |  |  | 1 | | 0.0 |
|  | *Haemophilus* sp. |  |  | 3 | | 0.1 |
| **F. anaerobic / GNB** | ***Salmonella*** |  |  | **1** | | **0.0** |
|  | *Salmonella enterica* serogroup E |  |  | 1 | | 0.0 |
| **F. anaerobic / GNB** | ***Eikenellacorrodens*** |  |  | **27** | | **0.8** |
| **F. anaerobic / GNB** | ***Citrobacter*** | **3** | **8.6** | **529** | | **14.8** |
|  | *Citrobacter diversus* | 3 | 8.6 | 496 | | 13.9 |
|  | *Citrobacter freundii* |  |  | 26 | | 0.7 |
|  | *Citrobacter amalonaticus* |  |  | 7 | | 0.2 |
| **F. anaerobic / GPB** | ***Corynebacterium*** | **1** | **2.9** | **178** | | **5.0** |
|  | *Corynebacterium accolens* |  |  | 6 | | 0.2 |
|  | *Corynebacterium amycolatum* |  |  | 3 | | 0.1 |
|  | *Corynebacterium propinquum* |  |  | 6 | | 0.2 |
|  | *Corynebacterium pseudodiphtheriticum* |  |  | 2 | | 0.1 |
|  | *Corynebacterium striatum* |  |  | 1 | | 0.0 |
|  | *Corynebacterium tuberculostearicum* |  |  | 2 | | 0.1 |
|  | *Corynebacterium jeikeium* |  |  | 6 | | 0.2 |
|  | *Corynebacterium* sp. | 1 | 2.9 | 152 | | 4.3 |
| **F. anaerobic / GNB** | ***Serratia marcescens*** |  |  | **45** | | **1.3** |
| **F. anaerobic / GNB** | ***Morganella morganii*** | **1** | **2.9** | **28** | | **0.8** |
| **F. anaerobic / GPB** | ***Actinomyces*** | **2** | **5.7** | **40** | | **1.1** |
|  | *Actinomyces naeslundii* |  |  | 1 | | 0.0 |
|  | *Actinomyces odontolyticus* |  |  | 8 | | 0.2 |
|  | *Actinomyce soris* |  |  | 2 | | 0.1 |
|  | *Actinomyces* sp. | 2 | 5.7 | 29 | | 0.8 |
| **F. anaerobic / GPB** | ***Lactobacillus*** |  |  | **3** | | **0.1** |
| **F. anaerobic / GNB** | ***Providencia*** |  |  | **3** | | **0.1** |
|  | *Providencia rettgeri* |  |  | 3 | | 0.1 |
| **F. anaerobic / GNB** | ***Aeromonas*** |  |  | **2** | | **0.1** |
|  | *Aeromonas hydrophila* |  |  | 2 | | 0.1 |
| **F. anaerobic / GPB** | ***Bacillus cereus*** |  |  | **2** | | **0.1** |
| **F. anaerobic / GNB** | ***Compylobacter*** |  |  | **1** | | **0.0** |
|  | *Campylobacter rectus* | 1 | 2.9 | 1 | | 0.0 |
| **F. anaerobic / GNB** | ***Hafnia alvei*** |  |  | **2** | | **0.1** |
| **F. anaerobic / GNB** | ***Panoea*** |  |  | **2** | | **0.1** |
|  | *Pantoea calida* |  |  | 1 | | 0.0 |
|  | *Pantoea dispersa* |  |  | 1 | | 0.0 |
| **F. anaerobic / GPC** | ***Peptoniphilus harei*** |  |  | **3** | | **0.1** |
| **F. anaerobic / GPC** | ***Morbillorum*** |  |  | **3** | | **0.1** |
|  |  |  |  |  | |  |
| **Aerobic / GNB** | ***Pseudomonas*** | **10** | **28.6** | **300** | | **8.4** |
|  | *Pseudomonas aeruginosa* | 10 | 28.6 | 284 | | 7.9 |
|  | *Pseudomonas aeruginosa*-CR^†^ |  |  | 2 | | 0.1 |
|  | *Pseudomonas otitidis* |  |  | 1 | | 0.0 |
|  | *Pseudomonas putida* group |  |  | 1 | | 0.0 |
|  | *Pseudomonas cepacia* |  |  | 1 | | 0.0 |
|  | *Pseudomonas mendocina* |  |  | 1 | | 0.0 |
|  | *Pseudomonas paucimobilis* |  |  | 1 | | 0.0 |
|  | *Pseudomonas stutzeri* |  |  | 1 | | 0.0 |
|  | *Pseudomonas diminuta* |  |  | 1 | | 0.0 |
|  | *Pseudomonas* sp. |  |  | 7 | | 0.2 |
| **Aerobic / GNB** | ***Acinetobacter*** | **1** | **2.9** | **38** | | **1.1** |
|  | *Acinetobacter baumannii* | 1 | 2.9 | 18 | | 0.5 |
|  | *Acinetobacter lwoffii* |  |  | 2 | | 0.1 |
|  | *Acinetobacter johnsonii* |  |  | 1 | | 0.0 |
|  | *Acinetobacter junii* |  |  | 5 | | 0.1 |
|  | *Acinetobacter ursingii* |  |  | 1 | | 0.0 |
|  | *Acinetobacter pittii* |  |  | 3 | | 0.1 |
|  | *Acinetobacter* sp. |  |  | 8 | | 0.2 |
| **Aerobic / GNC** | ***Neisseria*** | **1** | **2.9** | **40** | | **1.1** |
|  | *Neisseria meningitidis* |  |  | 1 | | 0.0 |
|  | *Neisseria mucosa* |  |  | 1 | | 0.0 |
|  | *Neisseria* sp. | 1 | 2.9 | 38 | | 1.1 |
| **Aerobic / GPC** | ***Stomatococcus*** |  |  | **3** | | **0.1** |
| **aerobic / GNB** | ***Stenotrophomonas maltophilia*** |  |  | **24** | | **0.7** |
| **aerobic / GPC** | ***Micrococcus*** |  |  | **2** | | **0.1** |
| **aerobic / GNB** | ***Moraxella*** |  |  | **46** | | **1.3** |
|  | *Moraxella catarrhalis* |  |  | 42 | | 1.2 |
|  | *Moraxella nonliquefaciens* |  |  | 2 | | 0.1 |
|  | *Moraxella* sp. |  |  | 2 | | 0.1 |
| **Aerobic /GPB** | ***Nontuberculous mycobacterium*** |  |  | **1** | | **0.0** |
| **Aerobic /GNB** | ***Kluyvera ascorbata*** |  |  | **1** | | **0.0** |
| **Aerobic /GNB** | ***Raoultella* sp.** |  |  | **1** | | **0.0** |
| **Aerobic /GPC** | ***Rothia mucilaginosa*** |  |  | **1** | | **0.0** |
| **Aerobic /GNB** | ***Burkholderia cepacia* complex** |  |  | **2** | | **0.1** |
| **Aerobic /GNB** | ***Elizabethkingia* sp.** |  |  | **1** | | **0.0** |
| **Aerobic / GNB** | ***Alcaligenes xylosoxidans*** |  |  | **1** | | **0.0** |
|  |  |  |  |  | |  |
| **Anaerobic / GPC** | ***Peptostreptococcus*** | **4** | **11.4** | **893** | | **25.0** |
|  | *Peptostreptococcus micros* | 3 | 8.6 | 299 | | 8.4 |
|  | *Peptostreptococcus anaerobius* |  |  | 32 | | 0.9 |
|  | *Peptostreptococcus magnus* |  |  | 156 | | 4.4 |
|  | *Peptostreptococcus* *asaccharolyticus* |  |  | 8 | | 0.2 |
|  | *Peptostreptococcus indolicus* |  |  | 1 | | 0.0 |
|  | *Peptostreptococcus* sp. | 1 | 2.9 | 397 | | 11.1 |
| **Anaerobic / GNB** | ***Prevotella*** | **2** | **5.7** | **462** | | **12.9** |
|  | *Prevotella disiens* |  |  | 2 | | 0.1 |
|  | *Prevotella bivia* |  |  | 8 | | 0.2 |
|  | *Prevotella buccae* |  |  | 23 | | 0.6 |
|  | *Prevotella intermedia* | 1 | 2.9 | 87 | | 2.4 |
|  | *Prevotella melaninogenica* |  |  | 58 | | 1.6 |
|  | *Prevotella denticola* |  |  | 3 | | 0.1 |
|  | *Prevotella oralis* |  |  | 9 | | 0.3 |
|  | *Prevotella oris* |  |  | 10 | | 0.3 |
|  | *Prevotella baroniae* |  |  | 5 | | 0.1 |
|  | *Prevotella histicola* |  |  | 4 | | 0.1 |
|  | *Prevotella nanceiensis* |  |  | 1 | | 0.0 |
|  | *Prevotella nigrescens* |  |  | 2 | | 0.1 |
|  | *Prevotella pallens* |  |  | 3 | | 0.1 |
|  | *Prevotella heparinolytica* |  |  | 1 | | 0.0 |
|  | *Prevotella loescheii* |  |  | 2 | | 0.1 |
|  | *Prevotella* sp. | 1 | 2.9 | 244 | | 6.8 |
| **Anaerobic / GNC** | ***Veillonella*** | **2** | **5.7** | **121** | | **3.4** |
|  | *Veillonella dispar* |  |  | 35 | | 1.0 |
|  | *Veillonella parvula* | 1 | 2.9 | 11 | | 0.3 |
|  | *Veillonella atypica* |  |  | 5 | | 0.1 |
|  | *Veillonella* sp. | 1 | 2.9 | 70 | | 2.0 |
| **Anaerobic / GNB** | ***Fusobacter*** | **4** | **11.4** | **250** | | **7.0** |
|  | *Fusobacter nucleatum* | 2 | 5.7 | 133 | | 3.7 |
|  | *Fusobacter necrophorum* |  |  | 14 | | 0.4 |
|  | *Fusobacterium mortiferum* |  |  | 1 | | 0.0 |
|  | *Fusobacterium naviforme* |  |  | 1 | | 0.0 |
|  | *Fusobacter varium* |  |  | 2 | | 0.1 |
|  | *Fusobacter* sp. | 2 | 5.7 | 99 | | 2.8 |
| **Anaerobic / GNB** | ***Bacteroides*** |  |  | **38** | | **1.1** |
|  | Bacteroides thetaiotaomicron |  |  | 7 | | 0.2 |
|  | *Bacteroides fragilis* |  |  | 13 | | 0.4 |
|  | *Bacteroides caccae* |  |  | 1 | | 0.0 |
|  | *Bacteroides gracilis* |  |  | 2 | | 0.1 |
|  | *Bacteroides ovatus* |  |  | 2 | | 0.1 |
|  | *Bacteroides uniformis* |  |  | 2 | | 0.1 |
|  | *Bacteroides vulgatus* |  |  | 2 | | 0.1 |
|  | *Bacteroides* sp. |  |  | 9 | | 0.3 |
| **Anaerobic / GPB** | ***Cutibacterium*** | **19** | **54.3** | **1925** | | **53.8** |
|  | *Cutibacterium acnes* | 9 | 25.7 | 981 | | 27.4 |
|  | *Cutibacterium granulosum* | 4 | 11.4 | 126 | | 3.5 |
|  | *Cutibacterium avidum* | 3 | 8.6 | 355 | | 9.9 |
|  | *Cutibacterium propionicus* |  |  | 2 | | 0.1 |
|  | *Cutibacterium* sp. | 3 | 8.6 | 461 | | 12.9 |
| **Anaerobic / GPB** | ***Clostridium*** |  |  | **9** | | **0.3** |
|  | *Clostridium sporogenes* |  |  | 1 | | 0.0 |
|  | *Clostridium perfringens* |  |  | 1 | | 0.0 |
|  | *Clostridium* sp. |  |  | 7 | | 0.2 |
| **Anaerobic / GNC** | ***Porphyromonas*** | **1** | **2.9** | **37** | | **1.0** |
|  | *Porphyromonas gingivalis* |  |  | 5 | | 0.1 |
|  | *Porphyromonas* sp. | 1 | 2.9 | 32 | | 0.9 |
| **Anaerobic / GNB** | ***Capnocytopha*ga** |  |  | **3** | | **0.1** |
| **Anaerobic / GPC** | ***Anaerococcus*** |  |  | **7** | | **0.2** |
|  | *Anaerococcus octavius* |  |  | 5 | | 0.1 |
|  | *Anaerococcus* sp. |  |  | 2 | | 0.1 |
| **Anaerobic / GPB** | ***Bulleidia extructa*** |  |  | **1** | | **0.0** |
| **Anaerobic / GPB** | ***Bifidobacterium longum*** |  |  | **1** | | **0.0** |
| **Anaerobic / GNC** | ***Dialister pneumosintes*** |  |  | **12** | | **0.3** |
| **Anaerobic / GPB** | ***Eggerthia catenaformis*** |  |  | **1** | | **0.0** |
| **Anaerobic / GPB** | ***Lachnoanaerobaculum saburreum*** |  |  | **1** | | **0.0** |
| **Anaerobic / GNB** | ***Leptotrichia* sp.** |  |  | **1** | | **0.0** |
| **Anaerobic / GNC** | ***Leuconostoc*** |  |  | **1** | | **0.0** |
| **Anaerobic / GNC** | ***Megasphaera micronuciformis*** |  |  | **2** | | **0.1** |
| **Anaerobic / GNB** | ***Slackiaexigua*** |  |  | **12** | | **0.3** |
| **Anaerobic / GNB** | ***Tissierella praeacuta*** |  |  | **1** | | **0.0** |
| **Anaerobic / GPB** | ***Atopobium parvulum*** |  |  | **1** | | **0.0** |
| **Anaerobic / GPC** | **Gm(+) cocci** |  |  | **1** | | **0.0** |
|  |  |  |  |  | |  |
| Fungus | Yeast-like | 1 | 1.1 | 15 | | 0.4 |
|  | Mold |  |  | 31 | | 0.9 |
|  | ***Candida*** |  |  | **19** | | **0.5** |
|  | *Candida albicans* |  |  | 7 | | 0.2 |
|  | *Candida famata* |  |  | 1 | | 0.0 |
|  | *Candida metapsilosis* |  |  | 1 | | 0.0 |
|  | *Candida parapsilosis* |  |  | 5 | | 0.1 |
|  | *Candida tropicalis* |  |  | 1 | | 0.0 |
|  | *Candida* sp. |  |  | 4 | | 0.1 |
|  | *Penicillium* sp. |  |  | 1 | | 0.0 |
|  | *Aspergillus* sp. |  |  | 28 | | 0.8 |
|  | *Chrysonilia* |  |  | 1 | | 0.0 |
|  | *Curvularia* sp. |  |  | 1 | | 0.0 |
|  | *Debaryomyce shansenii* |  |  | 1 | | 0.0 |
|  | *Exophiala* sp. |  |  | 2 | | 0.1 |
|  | *Fusarium* sp. |  |  | 1 | | 0.0 |
|  | *Kodamaea ohmeri* |  |  | 1 | | 0.0 |
|  | *Paecilomyces* sp. |  |  | 1 | | 0.0 |
|  | *Pseudallescheria boydii* |  |  | 4 | | 0.1 |
|  | *Rhodotorula* sp. |  |  | 2 | | 0.1 |
|  | *Scytalidium* |  |  | 1 | | 0.0 |
|  | *Trichophyton* sp. |  |  | 4 | | 0.1 |
|  | *Trichosporon* sp. |  |  | 1 | | 0.0 |
|  | *Verticillium* |  |  | 1 | | 0.0 |
|  |  |  |  |  | |  |
| **Unknown** | Unknown GPB |  |  | 3 | | 0.1 |
|  | Unknown GNB |  |  | 2 | | 0.1 |
|  | Unknown_Aerobes |  |  | 40 | |  |

sp.: species

^*^F. anaerobic: facultative anaerobic

^#^ESBL: extended-spectrum β-lactamases

^†^CR: Carbapenem-resistant
