# Supplementary material for: The difference in pathogenic bacteria between chronic rhinosinusitis in patients with and without Sjogren’s syndrome: a retrospective case–control study
Source: BMC Infect Dis. 2022 Aug 2;22:666. doi: 10.1186/s12879-022-07652-4 (PMC9344658; doi:10.1186/s12879-022-07652-4)
Supplement: Supplementary file 2 — Additional file 2: Table S2. Demographic characteristics in bacterial growth of SS-CRS and non-SS-CRS. [file 12879_2022_7652_MOESM2_ESM.docx]

**Additional Table S2. Demographic characteristics in bacterial growth of SS-CRS and non-SS-CRS.**

| Variables | SS-CRS | | non-SS-CRS | |  |
| --- | --- | --- | --- | --- | --- |
|  | N = 35 | | N = 3575 | | *p*-value |
|  | n | % | n | % |  |
| Gender |  |  |  |  | <0.001 |
| Male | 9 | 25.7 | 2107 | 58.9 |  |
| Female | 26 | 74.3 | 1468 | 41.1 |  |
| Age (years) | | | | | 0.097 |
| <65 | 26 | 74.3 | 3021 | 84.5 |  |
| ≥65 | 9 | 25.7 | 554 | 15.5 |  |
| Covariates | | | | | |
| RA | 4 | 11.4 | 25 | 0.7 | <0.001 |
| DM | 6 | 17.1 | 435 | 12.2 | 0.431 |
| HTN | 21 | 60.0 | 831 | 23.2 | <0.001 |
| CKD | 5 | 14.3 | 104 | 2.9 | 0.004 |
| CVA | 5 | 14.3 | 160 | 4.5 | 0.020 |
| CAD | 6 | 17.1 | 163 | 4.6 | 0.005 |
| COPD | 6 | 17.1 | 219 | 6.1 | 0.019 |
| Asthma | 6 | 17.1 | 268 | 7.5 | 0.045 |
