# Supplementary material for: The difference in pathogenic bacteria between chronic rhinosinusitis in patients with and without Sjogren’s syndrome: a retrospective case–control study
Source: BMC Infect Dis. 2022 Aug 2;22:666. doi: 10.1186/s12879-022-07652-4 (PMC9344658; doi:10.1186/s12879-022-07652-4)
Supplement: Supplementary file 3 — Additional file 3: Table S3. The comparison of bacterial genera between SS-CRS and non-SS-CRS. [file 12879_2022_7652_MOESM3_ESM.doc]

**Additional Table S3. The comparison of bacterial genera between SS-CRS and non-SS-CRS.**

|  |  | **SS-CRS** | | **non-SS-CRS** | |  |  |
| --- | --- | --- | --- | --- | --- | --- | --- |
|  |  | N | % | N | % |  |  |
| **Num of cases** | | 156 |  | 14259 |  |  |  |
| Num of cultures | | 40 | 25.6 | 3920 | 27.5 |  |  |
| Num of bacterial growth | | 35 | 87.5 | 3575 | 91.2 |  |  |
| **Classification** |  | n | % | n | % | **power** | ***p*-value** |
| facultative anaerobic / GPC | *Staphylococcus* | 26 | 74.3 | 2373 | 66.4 | 0.15 | 0.324* |
| facultative anaerobic / GNB | *Klebsiella* | 5 | 14.3 | 564 | 15.8 | 0.05 | 0.810* |
| facultative anaerobic / GPC | *Streptococcus* | 7 | 20.0 | 692 | 19.4 | 0.05 | 0.924* |
| facultative anaerobic / GPC | *Enterococcus* | 1 | 2.9 | 29 | 0.8 | 0.06 | 0.254 |
| facultative anaerobic / GNB | *Enterobacter* | 2 | 5.7 | 350 | 9.8 | 0.09 | 0.574 |
| facultative anaerobic / GNB | E*.coli* | 1 | 2.9 | 104 | 2.9 | <0.05 | 1.000 |
| facultative anaerobic / GNB | *Proteus* | 1 | 2.9 | 76 | 2.1 | <0.05 | 0.532 |
| facultative anaerobic / GNB | *Citrobacter* | 3 | 8.6 | 528 | 14.8 | 0.14 | 0.469 |
| facultative anaerobic / GPB | *Corynebacterium* | 1 | 2.9 | 178 | 5.0 | 0.05 | 1.000 |
| facultative anaerobic / GNB | *Morganella morganii* | 1 | 2.9 | 28 | 0.8 | 0.07 | 0.247 |
| facultative anaerobic / GPB | *Actinomyces* | 2 | 5.7 | 40 | 1.1 | 0.22 | 0.062 |
| aerobic / GNB | *Pseudomonas* | 10 | 28.6 | 298 | 8.3 | 0.85 | 0.001 |
| aerobic / GNB | *Acinetobacter* | 1 | 2.9 | 36 | 1.0 | 0.06 | 0.304 |
| aerobic / GNC | *Neisseria* | 1 | 2.9 | 40 | 1.1 | 0.05 | 0.331 |
| anaerobic / GPC | *Peptostreptococcus* | 4 | 11.4 | 845 | 23.6 | 0.38 | 0.109 |
| anaerobic / GNB | *Prevotella* | 2 | 5.7 | 415 | 11.6 | 0.15 | 0.424 |
| anaerobic / GNC | *Veillonella* | 2 | 5.7 | 119 | 3.3 | 0.06 | 0.329 |
| anaerobic / GNB | *Fusobacter* | 4 | 11.4 | 245 | 6.9 | 0.10 | 0.300 |
| anaerobic / GPB | *Cutibacterium* | 13 | 37.1 | 1585 | 44.3 | 0.13 | 0.394* |
| anaerobic / GNC | *Porphyromonas* | 1 | 2.9 | 37 | 1.0 | 0.06 | 0.311 |
| fungus | Yeast-like | 1 | 2.9 | 15 | 0.4 | 0.10 | 0.145 |

The *p*-values with * were analyzed by using Pearson’s chi-square tests, and otherwise, Fisher’s exact tests were applied.
