# Supplementary figures and images for: The difference in pathogenic bacteria between chronic rhinosinusitis in patients with and without Sjogren’s syndrome: a retrospective case–control study
Source: BMC Infect Dis. 2022 Aug 2;22:666. doi: 10.1186/s12879-022-07652-4 (PMC9344658; doi:10.1186/s12879-022-07652-4)

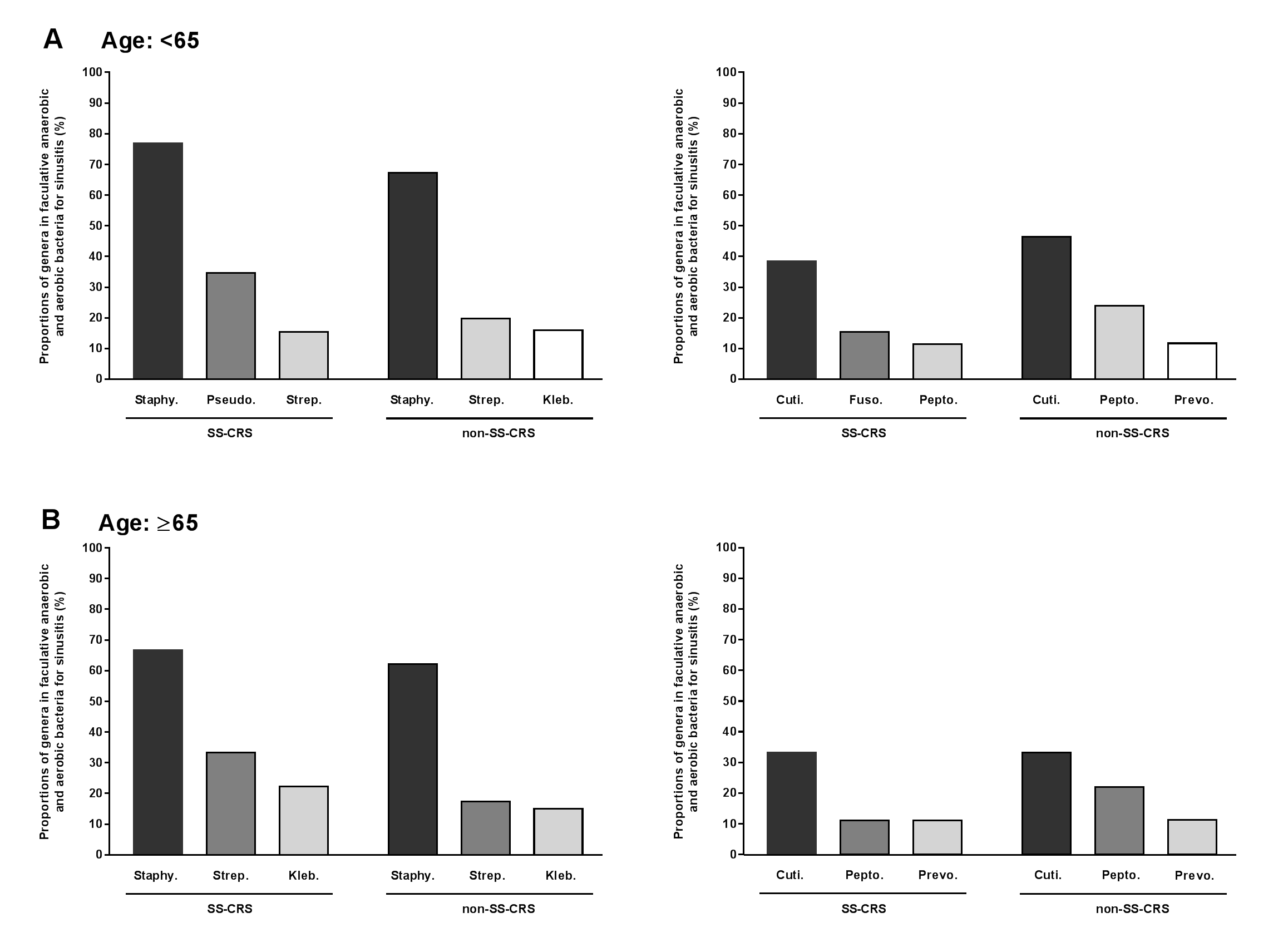

Supplement: Supplementary file 4 — Additional file 4. Figure S1. Top three bacterial genera of facultative anaerobes or aerobes and anaerobe in the age of SS-CRS and non-SS-CRS. [file 12879_2022_7652_MOESM4_ESM.tif]

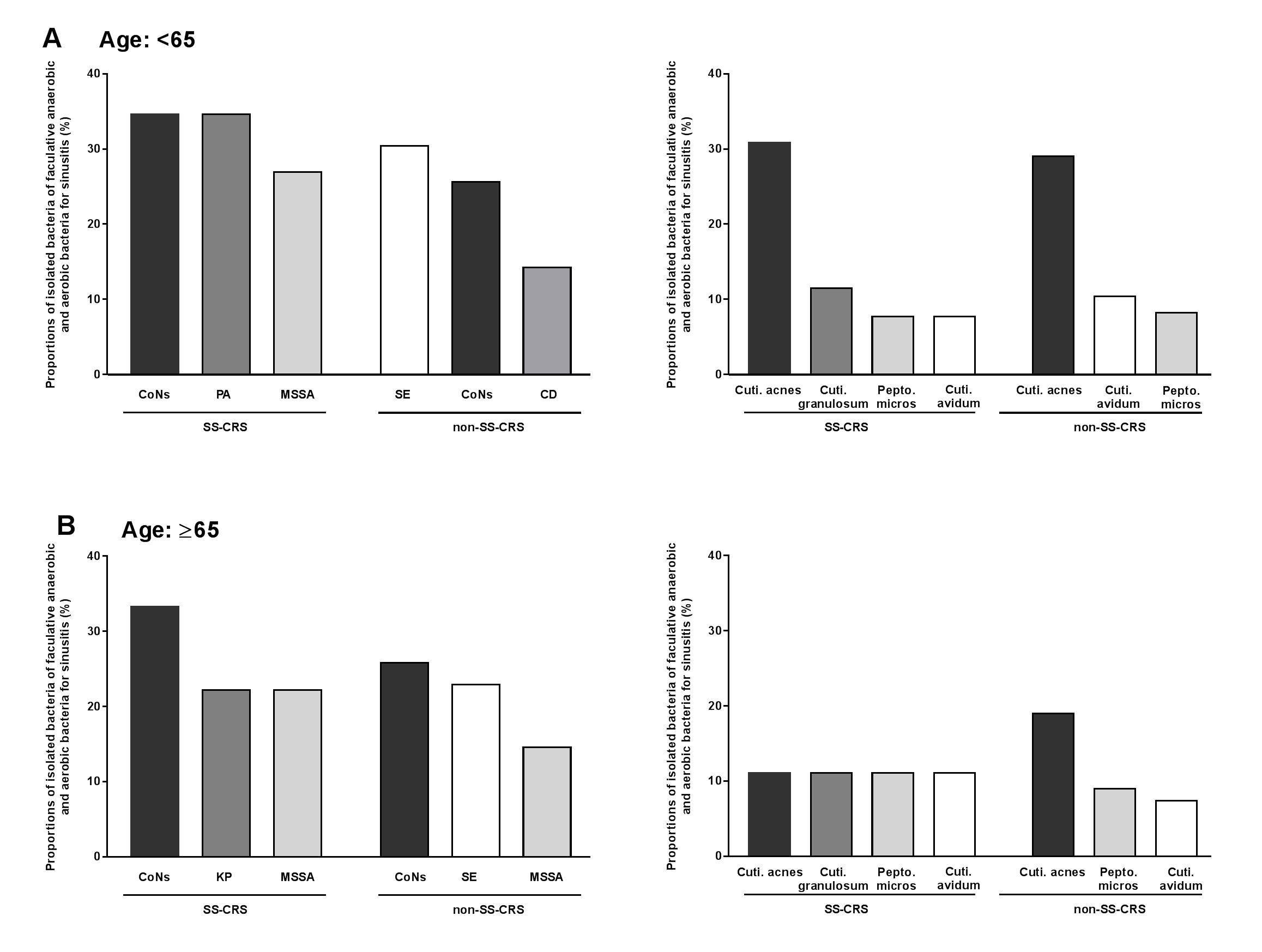

Supplement: Supplementary file 5 — Additional file 5. Figure S2. Top three isolated bacteria of facultative anaerobes or aerobes and anaerobe in the age of SS-CRS and non-SS-CRS. [file 12879_2022_7652_MOESM5_ESM.tif]
